# Carotid endarterectomy and blood-brain barrier permeability in subjects with bilateral carotid artery stenosis

**DOI:** 10.1186/s41016-025-00398-3

**Published:** 2025-06-17

**Authors:** Changyu Lu, Chenyu Zhu, Wenjie Li, Huan Zhu, Qihang Zhang, Tong Liu, Tongyu Yang, Yan Zhang

**Affiliations:** 1 Department of Neurosurgery, Peking University International Hospital, Beijing, China; 2 China National Clinical Research Center for Neurological Diseases, Beijing, China; 3 Department of Neurosurgery, Beijing Tiantan Hospital, Capital Medical University, Beijing, China

**Keywords:** Computed tomography perfusion, Carotid endarterectomy, Carotid artery stenosis, Blood–brain barrier, Permeability surface

## Abstract

**Background:**

The increased permeability of the blood–brain barrier (BBB) is related to the occurrence and development of diseases such as acute ischemic stroke, chronic ischemia, or small vessel disease. Patients with carotid artery stenosis have chronic ischemia. The exact effect of carotid endarterectomy on the blood–brain barrier is still unclear. The aim of the study was to assess the effect of carotid endarterectomy on basic perfusion parameters and permeability surface area-product (PS).

**Methods:**

The study included a total of 17 subjects (13 men), of which bilateral carotid artery stenosis was greater than 70%. All patients underwent unilateral carotid endarterectomy. Differences in the following computed tomography perfusion (CTP) parameters were compared before and after operation: cerebral blood flow (CBF), cerebral blood volume (CBV), mean transit time (MTT), time to peak (TTP), and PS. PS acquired by CTP is used to measure the permeability of the BBB to contrast material.

**Results:**

Before surgery, the operative side exhibited significantly lower CBF (*p* = 0.001) and prolonged MTT (*p* = 0.002) and TTP (*p* = 0.001) compared to the nonoperative side, while PS and CBV showed no significant differences. After carotid endarterectomy, only the operative side demonstrated improvements, with CBV increasing by 9.4%, MTT decreasing by 20.3%, TTP decreasing by 14.1%, and PS decreasing by 27.5% (all *p* < 0.01). No significant changes were observed on the nonoperative side.

**Conclusions:**

Carotid endarterectomy augmented BBB permeability can be controlled by carotid endarterectomy in patients with carotid artery stenosis.

## Background

Carotid artery stenosis may result in chronic cerebral hypoperfusion, thereby initiating progressive white matter injury [1]. This pathophysiological process is characterized by the sequential disruption of tight junctions and the extracellular matrix, ultimately compromising the structural and functional integrity of the blood–brain barrier (BBB) [1].

Carotid endarterectomy (CEA) has been shown to partially reverse cognitive deficits in patients with carotid artery stenosis [2]. This phenomenon is hypothesized to arise from the restoration of cerebral perfusion, which may facilitate the recovery of neuronal metabolism and the re-expression of downregulated neurotransmitter receptors within cortical structures [3, 4]. Under pathological states such as ischemic stroke, BBB disruption permits the extravasation of plasma components into the brain parenchyma, exacerbating neuroinflammation and impairing neuronal function [5]. Whether similar alterations in chronic carotid stenosis are reversible, following surgical revascularization, remains to be elucidated.

The BBB plays a pivotal role in maintaining CNS homeostasis by tightly regulating the exchange of solutes and cells between the systemic circulation and the neural environment [6]. In physiological states, it is impermeable to iodinated contrast agents [7], thus enabling the application of computed tomography perfusion (CTP) as a noninvasive modality to quantify BBB function in vivo [8–11]. Among the derived parameters, permeability surface area-product (PS) is considered a proxy of BBB permeability, while CBF, CBV, MTT, and TTP collectively reflect cerebral hemodynamic status.

In contrast to prior investigations such as that of Szarmach et al. [12], which focused on patients with unilateral carotid artery stenosis and largely preserved collateral circulation, the present study enrolled patients with severe bilateral internal carotid artery stenosis (≥ 70% bilaterally). This cohort presents with globally impaired cerebral perfusion, allowing us to minimize confounding from hemispheric compensation. Accordingly, the objective of this study was to evaluate alterations in BBB permeability—quantified via PS on CTP—following CEA in this distinct population. Furthermore, we discuss the potential implications of BBB stabilization for long-term cognitive recovery. Emerging evidence has underscored the importance of BBB integrity in cognitive function recovery, particularly in the contexts of neurodegenerative disease, cerebral ischemia, and postinfectious neuroinflammation [13–15].

## Methods

### Patient population

During 2018–2020, patients (4 females and 13 males; mean age of 58.5 ± *SD* 8.78 years) who underwent a carotid endarterectomy in the Department of Neurosurgery at Beijing Tiantan Hospital were diagnosed with severe stenosis of bilateral carotid arteries based on carotid ultrasounds. The choice of surgical side was based on TTP results. The side with severe ischemia will be considered for surgery. This study was approved by the Institutional Review Boards (IRB) of the Beijing Tiantan Hospital (approval number: KYSQ2018-156–01). All medical images and clinical data were fully anonymized. All volunteers gave written informed consent to participate in the study.

A general examination was performed prior to the study; subjects with an abnormal blood pressure, hematocrit ratio, or heart rate values were excluded from the study. Subjects with renal insufficiency, uncontrolled hyperthyroidism, and hypersensitivity to iodine or history of adverse effects following the administration of contrast agents were also excluded from the study.

### Angiography

Cerebral computed tomography angiography was performed prior to carotid endarterectomy(CEA). Patients were considered for this study when the bilateral internal carotid artery stenosis exceeded 70% (using North American Symptomatic Carotid Endarterectomy Trial method) [16] (Buchan, 1991).

### Imaging protocol

All CTPs were performed on a 256-section multidetector CT scanner (Revolution CT, GE Healthcare, Milwaukee, USA) which included axial non-contrasted CT and perfusion CT scanning.

Two CTP examinations were carried out: the first was performed 1 week before the carotid endarterectomy procedure, while the second was performed months (7.2 ± 5.7) after carotid endarterectomy.

The NCCT parameters were 120 kVp, 300 mAs, 512 × 512 image matrix, 1-s rotation, 5-mm section thickness, and 5-mm intersection space, and CDTIvol was approximately 40 mGy.

CT perfusion was performed with the injection of 50 mL of iodinated contrast material (iohexol [Omnipaque], 350 mg of iodine per milliliter; GE Healthcare, Shanghai, China) into an antecubital vein at a rate of 6 mL/s by a contrast injector (missouri-XD2001, Ulrich, Germany), followed by 20 mL of saline flush at the same injection rate.

The acquisition parameters were set as follows: 80 kVp, 150 mAs, DFOV 25 cm, 1-s rotation, and 5-mm slice thickness at 0-mm interval. The total of 512 slices was obtained with a total scan time of 44 s. The CDTIvol was approximately 80 mGy.

All acquisition parameters were applied as instructed by the manufacturer for the brain perfusion study.

### Image post-processing

The raw datasets were loaded onto a dedicated diagnostic workstation (AW 4 GE Healthcare Technologies, Milwaukee, WI, USA) equipped with a professional post-processing software package to generate color overlay maps of dynamic cerebral enhancement data (CT Perfusion version 4D, GE Healthcare Technologies, Milwaukee, WI, USA).

MTT characterizes the average time of contrast agent residence within the tissue. In mathematical terms, the mean transit time is computed as the time between the initial impulse and the time of arrival. MTT is computed and displayed in seconds.

CBF is derived from the initial value of the impulse residue function and is computed and displayed in milliliters per 100 g of wet tissue per minute.

CBV is computed and displayed in milliliters per 100 g of wet tissue. The blood volume is the product of the blood flow and the mean transit time: *CBV* = CBF × MTT.

TTP is the time between the onset of the enhancement transient (last pre-enhancement image) and the peak value of the time curve (image with the maximum value before the first post-enhancement image). Time to peak is computed and displayed in seconds, using the raw time curve data directly.

PS is computed and displayed in milliliters per 100 g of wet tissue per minute. It is computed from the impulse residue function. Contrast agent diffusion appears in the impulse residue function as a residual enhancement that occurs after the initial impulse response and decreases exponentially with time.

**Fig. 1:**
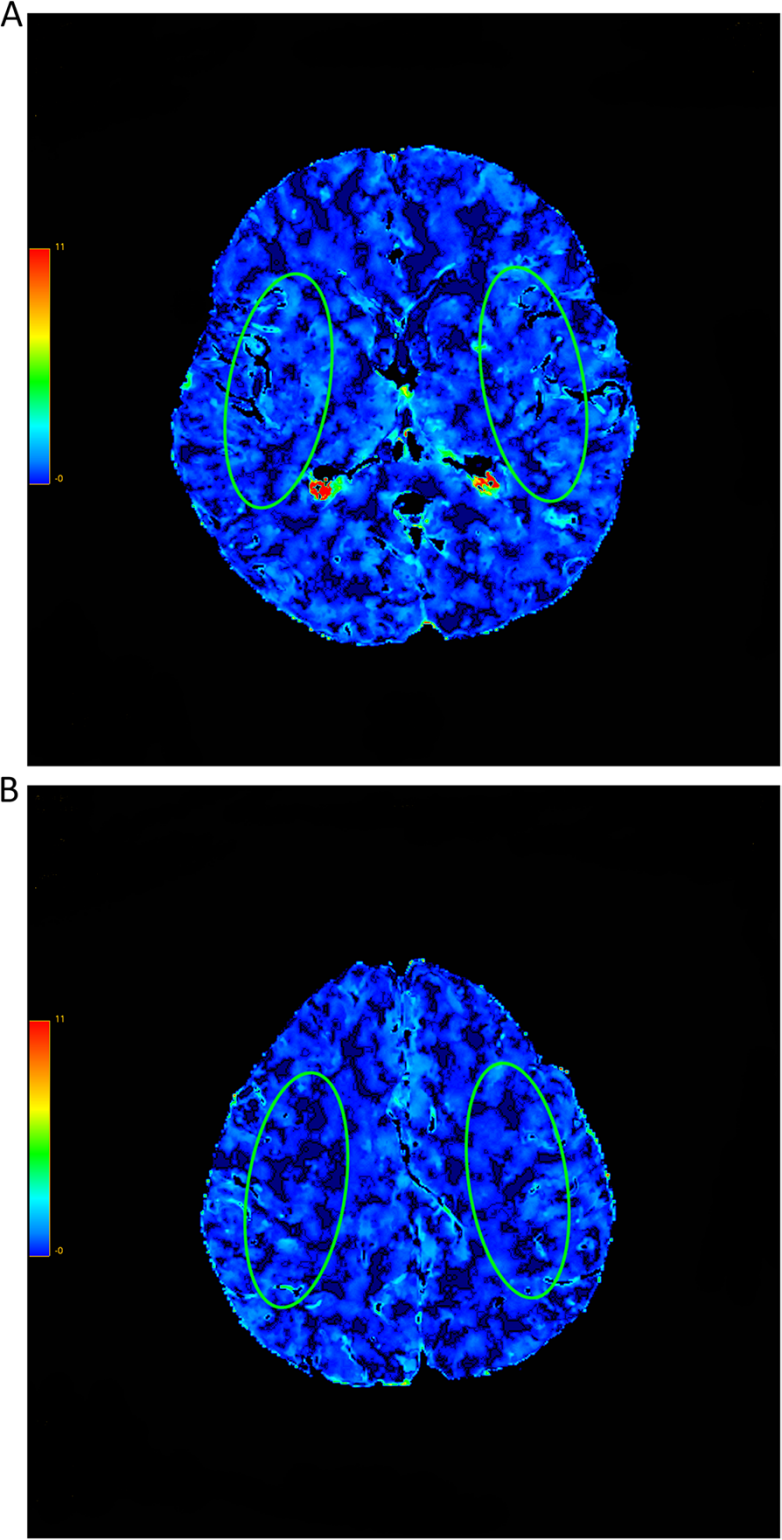
CT (computed tomography) perfusion color maps before carotid endarterectomy. Permeability surface product area (PS). Regions of interest (ROIs) placed on MCA (middle cerebral artery) territories. **A** The first slice of all CTP and **B** the last slice of all CTP

Blind to clinical information (including medical history, operative side, and time of surgery), two experienced neuroradiologists independently and manually delineated two standardized elliptical, mirrored regions of interest (ROIs). ROI placement was guided by anatomical landmarks, specifically at the junction of cortical gray matter and subcortical white matter within the centrum semiovale (Fig. 1). Each ROI (approximately 12 cm[2]) was positioned on all analyzed slices (Fig. 1A, B), over the cortical gray matter centered 20 mm from the edge of the brain. The large vessels were automatically excluded via brain perfusion software.

The absolute values of CT perfusion parameters (MTT, TTP, CBF, CBV, and PS) of one hemisphere in the region of middle cerebral artery distribution and contralateral mirroring areas in functional maps were measured.

### Statistical analysis

To compare results before and after carotid endarterectomy. Differences between the perfusion methods were assessed using only the parametric *t*-test. The level of significance was set at *α* = 0.05. All calculated *p*-values were for two-tailed tests. All statistical analyses were performed using SPSS 26.0 software (IBM SPSS, Inc., Chicago, IL, USA).

## Results

### Patient characteristics

A total of 17 patients (13 males and 4 females; mean age 58.5 ± 8.78 years) with bilateral internal carotid artery stenosis exceeding 70% were enrolled. All patients underwent unilateral carotid endarterectomy based on preoperative hemodynamic assessments.

### Computed tomography parameters before carotid endarterectomy

Prior to surgery, comparison between the operative and nonoperative hemispheres revealed significant hemodynamic differences. Cerebral blood flow (CBF) was significantly lower on the operative side (20.96 ± 2.32 vs 24.82 ± 3.43 mL/100 g/min, *p* = 0.001). Mean transit time (MTT) was significantly prolonged (8.74 ± 2.30 vs 6.80 ± 1.15 s, *p* = 0.002), and time to peak (TTP) was delayed (13.50 ± 1.81 vs 11.64 ± 1.03 s, *p* = 0.001). However, no significant differences were observed for cerebral blood volume (*CBV*: 2.27 ± 0.44 vs 2.08 ± 0.29 mL/100 g, *p* = 0.137) or permeability surface area-product (*PS*: 0.589 ± 0.156 vs 0.518 ± 0.122 mL/100 g/min, *p* = 0.152) between the two sides (Table 1).

**Table 1:** CT perfusion parameter values before carotid endarterectomy

Brain hemisphere	Independent samples *t*-test							
	*CT*	*Mean opera*	*Mean contra*	*t*	*No*	*p*-value	*SD opera*	*SD contra*
Operative side-before vs contralateral side-before	PS	0.589	0.518	1.469	17	0.152	0.156	0.122
Operative side-before vs contralateral side-before	CBV	2.273	2.078	1.524	17	0.137	0.440	0.290
Operative side-before vs. contralateral side-before	CBF	20.962	24.822	−3.847	17	0.001	2.319	3.426
Operative side-before vs. contralateral side-before	MTT	8.741	6.799	3.434	17	0.002	2.300	1.147
Operative side-before vs. contralateral side-before	TTP	13.501	11.636	3.668	17	0.001	1.812	1.031

*PS* permeability surface area-product, *CBV* cerebral blood volume, *CBF* cerebral blood flow, *MTT* mean transit time, *TTP* time to peak, *SD* standard deviation

### Computed tomography parameters after carotid endarterectomy

**Fig. 2:**
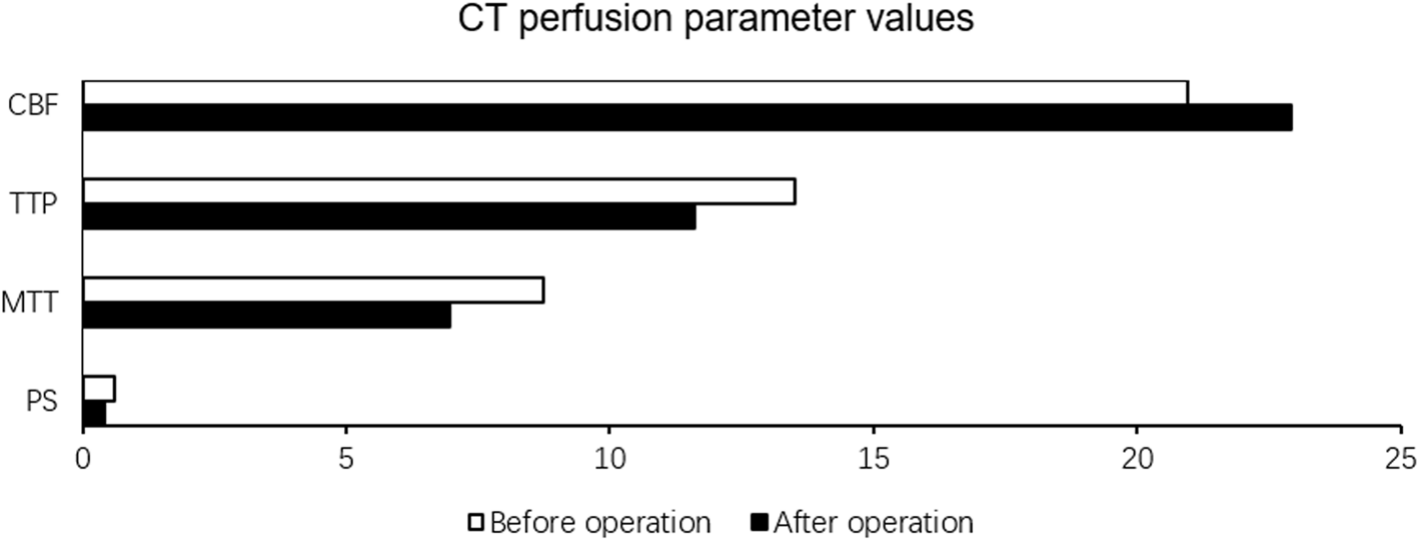
CT (computed tomography) perfusion parameter before and after operation

After carotid endarterectomy, significant improvements were observed exclusively in the operative hemisphere. CBV decreased from 2.27 ± 0.44 to 1.93 ± 0.45 mL/100 g (*p* = 0.006), MTT decreased from 8.74 ± 2.03 to 6.97 ± 1.13 s (*p* = 0.003), and TTP decreased from 13.50 ± 1.81 to 11.60 ± 1.35 s (*p* = 0.001). Importantly, PS declined significantly from 0.589 ± 0.156 to 0.427 ± 0.153 mL/100g/min (*p* = 0.007), indicating a reduction in BBB permeability. In contrast, no statistically significant changes were detected in CBF, CBV, MTT, TTP, or PS on the nonoperative side after surgery (all *p* > 0.05) (Table 2, Fig. 2).

**Table 2:** CT perfusion parameter values before and after carotid endarterectomy

Variable	Paired samples *t*-test					
	***Side***	***Mean***	***SD***	***No***	***t***	*p*-value
PS-1 PS-2	Operative	0.589 0.427	0.156 0.153	17	3.122	0.007
PS-1 PS-2	Non-operative	0.518 0.430	0.122 0.160	17	1.670	0.114
CBV-1 CBV-2	Operative	2.273 1.933	0.440 0.454	17	3.149	0.006
CBV-1 CBV-2	Non-operative	2.078 1.936	0.290 0.452	17	1.268	0.223
CBF-1 CBF-2	Operative	20.962 22.929	2.319 6.300	17	−1.200	0.248
CBF-1 CBF-2	Non-operative	24.822 24.521	3.426 6.367	17	0.194	0.849
MTT-1 MTT-2	Operative	8.741 6.967	2.030 1.128	17	3.523	0.003
MTT-1 MTT-2	Non-operative	6.799 6.464	1.147 1.083	17	0.975	0.344
TTP-1 TTP-2	Operative	13.501 11.602	1.812 1.354	17	3.933	0.001
TTP-1 TTP-2	Non-operative	11.636 11.179	1.031 1.327	17	1.868	0.080

*PS* permeability surface area-product, *CBV* cerebral blood volume, *CBF* cerebral blood flow, *MTT* mean transit time, *TTP* time to peak, *SD* standard deviation

## Discussion

There are two main findings of this study: (1) Carotid endarterectomy diminishes the increased BBB permeability in subjects with chronic artery stenosis, and (2) such decline is not found on the nonoperative hemisphere.

The choice of the surgical side is to choose the side with poor cerebral blood perfusion, which is determined according to the TTP. However, in our study, there are also differences in CBF and MTT between the operative side and the nonoperative side. Subjects with reduced CBF and cerebrovascular reactivity may include two different conditions: misery perfusion (or stage II ischemia) attributable to hemodynamic compromise and matched deteriorated metabolism attributable to incomplete infarction. Having said this, under hypoxic conditions, the non-infarcted tissue is present as a consequence of a combination of reduced perfusion and moderately reduced oxygen metabolism [17]. So, there will be more severe misery perfusion on the operative side. CBF was slightly lower, and MTT was higher on the operative side, which suggested that the collateral circulation of the operative side and the nonoperative side was incomplete. There are advantages for us to analyze the changes of PS after operation. Studies have shown that the CBV of some patients after carotid artery stent implantation is still not improved, so it is not surprising that there is no difference between the CBV of the surgical side and the nonoperative side when the bilateral collateral circulation is incomplete.

Previous studies have demonstrated that BBB permeability tends to increase during the acute phase of ischemic stroke [9]. Nonetheless, Horsch et al. [18] found no significant interhemispheric differences in PS among stroke patients, indicating a diffuse pattern of BBB disruption. Similarly, Szarmach et al. [12] reported no significant differences in PS between hemispheres in patients with unilateral carotid artery stenosis, a finding potentially attributable to effective collateral circulation mitigating regional ischemia. However, it is important to emphasize that our study population differs fundamentally from that of Szarmach et al. All patients enrolled herein exhibited severe bilateral internal carotid artery stenosis (≥ 70% bilaterally), leading to globally compromised cerebral perfusion and markedly limited collateral flow capacity. Consequently, the recovery of blood flow following CEA on the operative side could not significantly influence hemodynamic parameters on the contralateral nonoperative side. This lack of cross-hemispheric improvement explains why a significant postoperative decline in PS was observed exclusively on the operative side, while the nonoperative side exhibited no corresponding changes. Thus, our findings not only align with prior observations of PS homogeneity in chronic cerebrovascular disease but also challenge the generalizability of Szarmach et al.’s conclusions to populations with bilateral critical stenosis.

In our study, we found that CBV, MTT, and TTP of the surgical side were significantly improved after carotid endarterectomy. However, there was no statistical significance in the nonsurgical side, which also verified the incomplete collateral circulation in patients with bilateral carotid stenosis. The changes of the operative side were consistent with the previous studies. CBV have no change after blood flow recovery in most studies [15, 19, 20]. CBV increased after blood flow recovery in our study. The recovery of blood flow on the operative side is not helpful to the recovery of blood flow on the nonoperative side for patients with bilateral carotid stenosis. Lack of any interdependence between perfusion parameters (CBF, CBV, MTT, and TTP) and PS underlies the multifactorial nature of BBB breakdown encompassing deteriorated perfusion and an extensive inflammatory state [21]. Our study suggests that PS decreased on the operative side after carotid endarterectomy, which proved that the permeability of the blood–brain barrier decreased after carotid endarterectomy. All patients in our study had bilateral carotid stenosis of more than 70% and had neurological symptoms (dizziness, headache, visual impairment, and dyskinesia). Some studies have limitations because of patients who had unilateral carotid artery stenosis with good collateral circulation. Although the existence of collateral circulation is advantageous for blood perfusion, it is disadvantageous for the research results. To the best of our knowledge, we are the first to demonstrate that enhanced BBB permeability can be reduced after carotid endarterectomy in patients with bilateral carotid stenosis.

Quantitative CTP data is highly dependent on the post-processing software. Software differences are frequently considered the main cause of variability in perfusion results [22–24]. We use the same equipment and the same program for post-processing to improve the reliability of our data. In addition, our data were drawn at the level and location of the middle cerebral artery blood supply area by two experienced radiologists. Finally, we selected eight layers to draw the ROI (an area of approximately 10 cm[2] each) and calculate the perfusion parameters. Importantly, in Johnson and Wilson model (this technique has been used in our investigations), PS and CBV are independent parameters. MTT, CBV, and CBF are calculated with the first phase of impulse residue function (IRF), and PS is calculated with the second phase of IRF [25]. Therefore, PS was not influenced by elevated CBV. This method has a wide application prospect and provides a simple and easy method for obtaining morphological parameters and perfusion parameters quickly and easily. Dynamic contrast-enhanced magnetic resonance imaging (MRI) has several advantages over CTP in BBB evaluation. It can be associated with blood oxygen level-dependent MRI to provide functional information about the effects of long-term ischemia and hypoxia on the central nervous system. It can be also associated with voxel-based morphometry. Our team will use dynamic contrast-enhanced MRI to do further study in patients with carotid stenosis.

This study has several limitations. First, the sample size was relatively small (*n* = 17), which may restrict the generalizability of our findings, although it is comparable to previous CTP-based BBB permeability studies, such as the work by Ivanidze J. et al. [26]. Second, the lack of long-term postoperative follow-up limits the evaluation of sustained effects. Third, collateral circulation was not directly assessed; however, given the severe bilateral carotid stenosis in our cohort, effective collateral flow was likely minimal, and its impact on BBB permeability measurements is considered negligible. Future studies with larger samples and extended follow-up are warranted.

## Conclusions

In this study, we enrolled patients with bilateral internal carotid artery stenosis, a population characterized by more extensive cerebral hypoperfusion compared to those with unilateral stenosis. Long-term cerebral ischemia is associated with increased BBB permeability. Our results suggest that CEA was associated with a reduction in BBB permeability on the operative side, whereas no significant changes were observed on the nonoperative side. This asymmetrical outcome may be related to the limited integrity of collateral circulation in cases of bilateral severe stenosis. To the best of our knowledge, this study is the first to demonstrate that CEA may help mitigate BBB permeability alterations in patients with bilateral carotid artery stenosis, providing more reliable evidence compared to studies focused on unilateral disease.

## Data Availability

The datasets generated for this study are available on request to the corresponding author.

## Abbreviations

PS: Permeability surface area-product
CTP: Computed tomography perfusion
CBF: Cerebral blood flow
CBV: Cerebral blood volume
MTT: Mean transit time
TTP: Time to peak
BBB: Blood-brain barrier
IRB: Institutional Review Board
CEA: Carotid endarterectomy
ROI: Regions of interest
IRF: Impulse residue function
MRI: Magnetic resonance imaging
